# Overexpression of matrix metalloproteinase‐9 in breast cancer cell lines remarkably increases the cell malignancy largely via activation of transforming growth factor beta/SMAD signalling

**DOI:** 10.1111/cpr.12633

**Published:** 2019-07-02

**Authors:** Haodi Dong, Hongxiu Diao, Ying Zhao, Huihao Xu, Shimin Pei, Jiafeng Gao, Jie Wang, Tariq Hussain, Deming Zhao, Xiangmei Zhou, Degui Lin

**Affiliations:** ^1^ The Clinical Department, College of Veterinary Medicine China Agricultural University Beijing China; ^2^ Key Laboratory of Animal Epidemiology of the Ministry of Agriculture and College of Veterinary Medicine China Agricultural University Beijing China

**Keywords:** canine breast cancer, invasion, malignancy, MMP‐9, TGF‐β/SMAD signalling

## Abstract

**Objectives:**

Matrix metalloproteinase 9 (MMP‐9) has been frequently noticed in the breast cancers. In this study, we aim to investigate the associations of MMP‐9 with the activation of transforming growth factor beta (TGF‐β)/SMAD signalling and the malignancy of breast malignant tumour cells.

**Materials and methods:**

The distributions of MMP‐9 and TGF‐β in the tissues of canine breast cancers were screened by immunohistochemical assays. A recombinant plasmid expressing mouse MMP‐9 was generated and transiently transfected into three different breast cancer cell lines. Cell Counting Kit‐8 and colony formation assay were used to study cell viability. Migration and invasion ability were analysed by wound assay and transwell filters. Western blot and quantitative real‐time PCR were used to determine the protein and mRNA expression.

**Result:**

Remarkable strong MMP‐9 and TGF‐β signals were observed in the malignant tissues of canine breast cancers. In the cultured three cell lines receiving recombinant plasmid expressing mouse MMP‐9, the cell malignancy was markedly increased, including the cell colony formation, migration and epithelial‐mesenchymal transition. The levels of activated TGF‐β, as well as SMAD4, SMAD2/3 and phosphorylation of SMAD2, were increased, reflecting an activation of TGF‐β/SMAD signalling. We also demonstrated that the inhibitors specific for MMP‐9 and TGF‐β sufficiently blocked the overexpressing MMP‐9 induced the activation of SMAD signalling and enhancement on invasion in the tested breast cancer cell lines.

**Conclusion:**

Overexpression of MMP‐9 increases the malignancy of breast cancer cell lines, largely via activation of the TGF‐β/SMAD signalling.

## 1 INTRODUCTION

Canine mammary carcinomas (CMCs) are the most common canine cancer, with an estimated annual incidence of 182 per 100 000 female dogs.^1^ The most common histological type is tubular carcinoma (adenocarcinoma), followed by papillary carcinoma, solid carcinoma, complex carcinoma and carcinosarcoma.^2^, ^3^ CMCs occur at elder ages, usually in the groups of 8‐10 years old.^2^ Despite unknown aetiology, several risk factors have been considered to be significantly associated, including hormonal, nutritional and genetic events.^2^, ^4^, ^5^, ^6^, ^7^ The prognosis of breast cancer depends on many clinical and pathological issues, such as clinical stage, histopathological pattern, tumour size, lymphatic or vascular invasion and distant metastases.^2^, ^4^, ^8^, ^9^ In the past decades, a number of cellular factors have been proposed to be potential for the prognosis of breast cancers, particularly human breast carcinomas, covering miRNAs, oncoproteins, mutations in several special genes, cancer stem cells and circulating tumour cells.^10^ Kaszak and the colleagues have reviewed the frequently addressed biomarkers of canine breast cancers, such as Ki‐67, PCNA and p53 for cancer cell proliferation and apoptosis, E‐cadherin, CEA and CA 15‐3 for metastatic potential and VEGF, EGFR and HER‐2 for angiogenesis. Because of the similarity between CMCs and human breast cancers, those biomarkers for humans may be also useful for the prognosis of CMCs.

Matrix metalloproteinases (MMPs) are a family of zinc‑dependent proteases; among them, MMP‑9 is one of the major members of MMP family.^11^, ^12^ MMP‑9 protein is mainly secreted by tumour cells and stromal cells, which usually present in the form of zymogen. Via the process of hydrolysis, the activated MMP‑9 is able to degrade basement membrane (BM) type IV collagen, which is believed to affect the ability of BMs to impede tumour cell movement.^13^ As type IV collagens are the main components of the extracellular matrix and BMs, tumour‐derived MMP‑9 may destroy these tissue barriers and enhance the invasion and metastasis of tumour cells. Actually, increased expression of MMP‐9 has been documented in many different histological types of human malignant tumours and their metastasis, such as oral, larynx, gastric, lung, liver, breast, bone, skin and cervical.^11^, ^13^, ^14^, ^15^, ^16^, ^17^, ^18^, ^19^ Therefore, MMP‐9 and its family members, such as MMP‐2, are considered as the prognostic biomarkers for various carcinomas.

Transforming growth factor beta (TGF‐β) is a multifunctional cytokine belonging to the transforming growth factor superfamily. Activated TGF‐β complexes, together with other factors, form a serine/threonine kinase complex that binds to TGF‐β receptors and further activate different downstream substrates and regulatory proteins, mainly the SMAD and DAXX pathways, inducing transcriptions of various target genes involving in cell differentiation, proliferation, chemotaxis and activation of many immune cells.^20^, ^21^ Increased expression of TGF‐β often correlates with the malignancy of many cancers. Activation of TGF‐β is induced by many elements, including pH, reactive oxygen species, thrombospondin‐1, protease and metalloprotease; among them, MMP‐9 and MMP‐2 are known to able to cleavage the latent TGF‐β.^22^ However, the exact association between overexpression of MMP‐9 and activation of SMAD pathway is still not clear.

In this study, we screened the expressions of MMP‐9 and TGF‐β in the samples of canine breast cancers and confirmed overexpression statues of those two proteins in the malignant tissues. Transient expression of mouse MMP‐9 in three different breast carcinoma cell lines increases the cell colony formation and migration and promotes epithelial‐mesenchymal transition (EMT). Apparent activation of SMAD signalling, represented by increased expressions of SMAD4, SMAD2/3 and phosphorylation of SMAD2, was noticed in the cell lines expressing MMP‐9. We also demonstrated that inhibition of the activity of either MMP‐9 or TGF‐β sufficiently blocked the MMP‐9 overexpression induced the activation of SMAD signalling and enhancement on invasion in the breast cancer cell lines.

## 2 MATERIALS AND METHODS

### 2.1 Ethics statement

All animal experiments were performed according to the Chinese Regulations of Laboratory Animals—The Guidelines for the Care of Laboratory Animals (Ministry of Science and Technology of People's Republic of China) and Laboratory Animal Requirements of Environment and Housing Facilities (GB 14925‐2010, National Laboratory Animal Standardization Technical Committee). The animal studies and research protocols were approved by The Laboratory Animal Ethical Committee of China Agricultural University under the licence number—20110611‐0.

### 2.2 Antibodies and reagents

Primary antibodies in this study include rabbit anti‐MMP‐9 antibody (Abcam), anti‐TGF‐beta 1 antibody (Abcam), anti‐SMAD4 polyclonal antibody (Proteintech), mouse anti‐SMAD2/3 antibody (Santa Cruz Biotechnology), rabbit anti‐phospho‐SMAD2 antibody (Cell Signaling Technology), mouse anti‐GAPDH antibody (Proteintech), rabbit anti‐E‐cadherin antibody (Proteintech), rabbit anti‐vimentin antibody (Proteintech), SB 431542 (Sigma‐Aldrich), GM6001 (Selleckchem) and TGF‐beta (Cell Signaling Technology). HRP‐goat anti‐mouse antibody and HRP‐goat anti‐rabbit antibody were purchased from Proteintech, Wuhan, China. PCMV‐HA plasmid and Lipofectamine 3000 reagent were purchased from Invitrogen, and MMP‐9‐PCMV‐HA was contracted by GenePharma. Eight‐micrometer pore‐size transwell filters for invasion assay were obtained from Costar, Corning Incorporated.

### 2.3 Cell culture

Mouse breast tumour cell line 4T1 (ATCC® CRL‐2539™) and human breast tumour cell line MDA‐MB‐231 (ATCC® HTB‐26™) were purchased from American Type Culture Collection (ATCC). Human breast tumour cell line MCF7 was obtained from Cell Bank of Chinese Academy of Science. Cell lines MCF7 and MDA‐MD‐231 were cultured in DMEM (Gibco, Life Technologies) with 10% foetal bovine serum (FBS, Gibco) and 100 µg/mL streptomycin and 100 U/mL penicillin (Gibco). Cell line 4T1 was cultured in RPMI‐1640 (Gibco) with 10% FBS and 100 µg/mL streptomycin and 100 U/mL penicillin. Cells were incubated at 37°C in a 5% CO_2_ environment.

### 2.4 Immunohistochemical (IHC) analysis

All the tumour tissues from canine were obtained from China Agricultural University Animal Hospital. Tumours were surgically dissected and fixed in 10% (v/v) neutral buffer formalin for 5 days. The fixed tissues were routinely dehydrated in ascending grades of ethanol and xylene, and then embedded in paraffin wax. Sections (5 μm) were prepared with microtome (Leica) and mounted on CITOGLAS ® adhesion microscope slides (CITOTEST).

### 2.5 Transfection

Extraction of the recombinant plasmid pMMP‐9‐HA DNA was performed with QIAGEN Plasmid Plus Kit (QIAGEN) according to the manufacturer's instruction. Cells were allowed to attach overnight in six‐well plates. 2.5 mg of the plasmid DNA was incubated with 3.75 μL (for 4T1 cells) or 7.5 μL (MDA‐MB‐231 and MCF7 cells) of Lipofectamine 3000 and 125 μL of Opti‐MEM medium at room temperature (RT) for 5 minutes and then transferred onto 4 × 10^5^ adherent cells. Cells were maintained with DMEM or RAPI‐1640 in the condition of 2% FBS 37°C for 48 hours before harvest.

### 2.6 Cell viability assay

Cell viability was measured with a commercial Cell Counting Kit‐8 (Beyotime). Briefly, the CCK‐8 solution was directly added to the cell wells and incubated at 37°C for 30 minutes. The absorbance at 450 nm (OD_450_) each well was obtained using a microplate reader with a background control as the blank. The cell viability was expressed as the percentage of the untreated control.

For the colony formation assay, properly resuspended cells were randomly plated in six‐well plate at a density of 1 × 10^4^ cells/well for 24 hours. 2.5 mg of the plasmid pMMP‐9‐HA was introduced into the cells with Lipofectamine 3000. After 10‐day incubation, cells were fixed by formalin and stained with 0.1% (W/V) crystal violet (Solarbio).

### 2.7 Migration assay

Wound assay was performed to evaluate the migration ability of cells. Cells were seeded in six‐well plate and grew to confluence followed by scratching the monolayer cells with a 200 μL pipette tip to create wound. Plates were washed to remove floating cells and debris, then transfected and photographed the cell migration images at 0, 24 and 48 hours post‐transfection. Each testing group contained at least three independent wells.

### 2.8 Invasion assay

Eight‐micrometer pore‐size transwell filters (Costar, Corning Incorporated) were put in 24‐well plate, and cells were seeded onto the filters at a concentration of 1 × 10^4^ cells/well in 100 μL of FBS‐free medium and then transfected with plasmid MMP‐9‐PCMV‐HA. The lower chambers were filled with 600 μL of medium with 10% FBS. TGF‐beta (50 pmol/L) or SB431542 (10 μmol/L) was added into each well 24 hours post‐transfection. Thirty‐six hours after treatment, cells on the topside of the filter were removed by scrubbing with a tipped swab. The migration of cells to the lower side of the filter was determined by crystal violet staining. Each testing group contained at least three independent wells.

### 2.9 Western Blot analysis

For the preparation of cell lysates, cells were washed twice with ice‐cold PBS and homogenized with RIPA buffer containing a cocktail of protease and phosphatase inhibitor (Sigma‐Aldrich) for 20 minutes on ice. Afterwards, samples were sonicated for 20 seconds and centrifuged at 12 000 *g* at 4°C for 20 minutes. The supernatants were collected and boiled for 10 minutes in loading buffer (250 nmol/L Tris‐HCl 6.8 pH, 10% sodium dodecyl sulphate, 0.5% bromophenol blue, 50% glycerol and 0.5 mol/L dithiothreitol). Equal amounts of protein were separated by 12% or 10% sodium dodecyl sulphate polyacrylamide gel electrophoresis (SDS‐PAGE) and transferred onto polyvinylidene difluoride membranes (Millipore). After blocking with 5% skim milk in Tris‐buffered Saline Tween‐20, membranes were incubated with the individual primary antibodies at 4°C overnight. Membranes were rinsed three times in TBST and then incubated with different HRP‐labelled secondary antibodies at 37°C for 60 minutes. Signals were developed using an enhanced chemiluminescence detection kit (Bio‐Rad).

### 2.10 Quantitative real‐time PCR

Total RNAs from cells were extracted with TRIzol reagent (Invitrogen) according to the manufacturer's instruction. The cDNA was synthesized with RevertAid First Strand cDNA Synthesis Kit (Thermo Fisher Scientific). The integrity and concentration of cDNA were measured by NanoDrop 2000 machine (Thermo Scientific). The expressions of the target genes were evaluated by qRT‐PCT on 700 Fast Real‐Time PCR Systems (ViiA7 Real‐time PCR, ABI), with AceQ qPCR SYBR Green Master Mix Kit (Vazyme Biotech). The primers of the different genes used in the present study are shown in Table 1. Standard PCR cycle parameters were as follows: 95°C for 300 seconds, followed by 40 cycles of 95°C for 10 seconds, 60°C for 30 seconds. The relative expression levels of mRNAs were determined by a comparative Ct (ΔΔ Ct) method.

**Table 1:** Primers used for quantitative real‐time PCR

Gene	Forward primer (5′‐3′)	Reverse primer (5′‐3′)
SMAD2	AGTGAGGAGCCAGGGGAGA	TTACAGCAAAGGTTGAGGAAGG
SMAD3	TCACCGACCCCTCCAATTC	GCCGCACGCCTCTTCC
SMAD4	CACTATGAGCGGGTTGTC	GGTGCTGGTGGCGTTAGA
GAPDH	CGACTTCAACAGCAACTCCCACTCTC	TGGGTGGTCCAGGGTTTCTTACTCCTT

### 2.11 Statistical analysis

Statistical comparisons between groups were performed using ANOVA (Prism GraphPad 5 Software). All data are presented as mean ± SD of three independent experiments. Significant differences were assigned at *P* < 0.05, <0.01 and <0.001, denoted by *, ** and ***, respectively.

## 3 RESULTS

### 3.1 Remarkably increased expressions of MMP‐9 and TGF‐beta in the tissues of canine breast cancers

**Figure 1:**
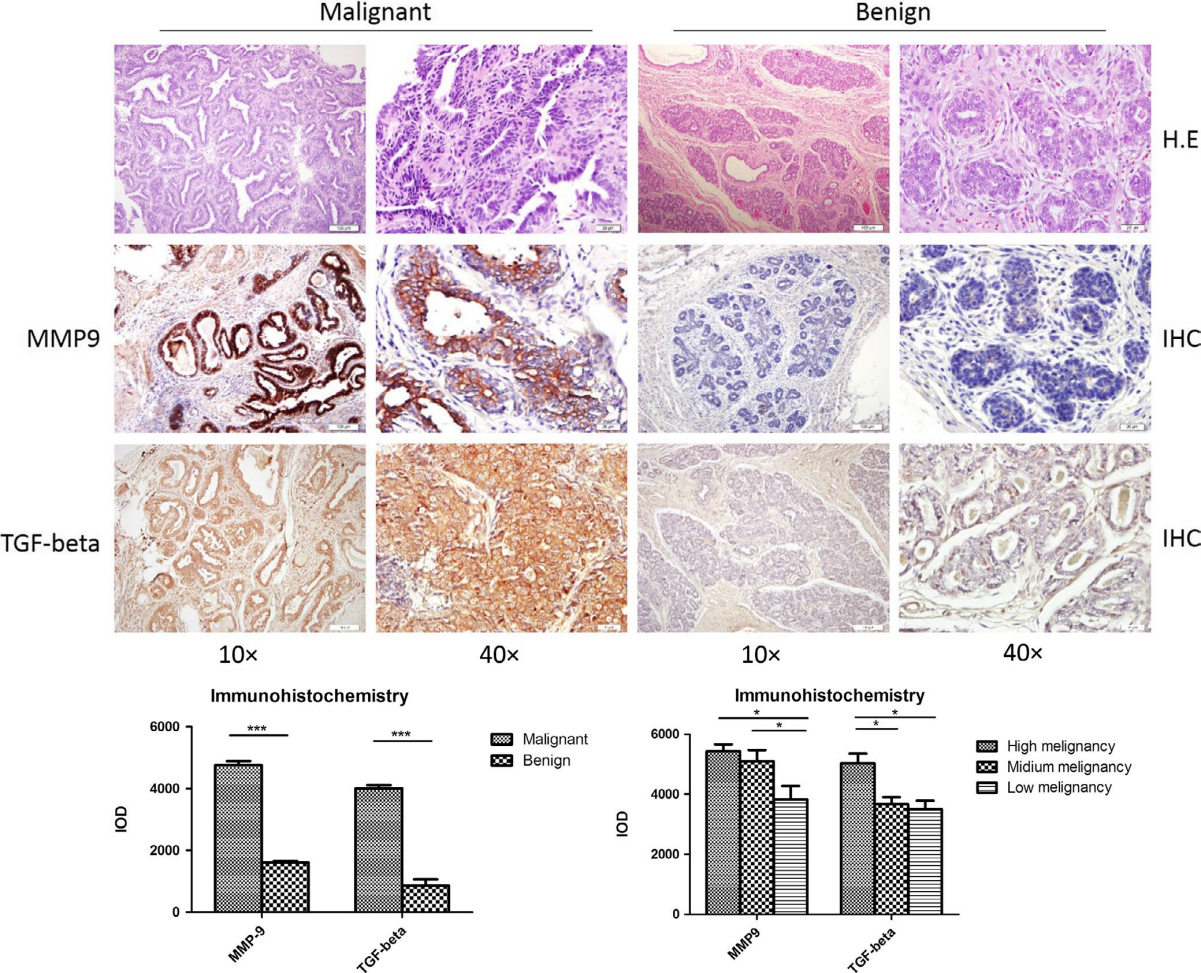
Histopathological assays of canine breast malignant and benign tumours. A, Pathological assays. Upper panel: representative images of HE stain; middle panel: representative images of matrix metalloproteinase (MMP)‐9‐specific immunohistochemical (IHC). Bottom panel: representative images of transforming growth factor beta (TGF‐β)‐specific IHC. The magnifications are shown. B, Quantitative assays of the average optical densities of MMP‐9 and TGF‐β signals in the malignant and benign tumours. Statistical analysis was performed using one‐way ANOVA. ***P < 0.001 and *P < 0.05 were considered significantly different

Increased expressions and distributions of MMP‐9 have been described in different malignant tissues of different hosts. To screen the distributions of MMP‐9 in canine breast cancers, the surgically removed samples from 34 cases of pathologically diagnosed tubulopapillary carcinoma characterized by the formation of tubules and/or papillary projects and 18 cases of benign breast adenoma with well‐differentiated luminal epithelial or myoepithelial cells were collected and enrolled in this study. MMP‐9 and TGF‐β expressions in breast carcinoma and adenoma were evaluated by the individual immunohistochemical (IHC) assays. As summarized in Table 2, markedly more cases of malignant tumours were MMP‐9 (85.3%, 29/34)‐ and TGF‐β (79.4%, 27/34)‐positive; among them, 24 cases were both MMP‐9‐ and TGF‐β‐positive. Large amounts of strongly brown‐stained MMP‐9 signals were detected in the malignant tissues, mostly surrousssssnding the carcinoma cells, whereas markedly less and weak positive signals in the sections of benign tumours (Figure 1A, middle panel). The brown‐stained fine signals of TGF‐β distributed widely in the tissues of malignant tumours, while almost unobservable in the benign ones (Figure 1A, bottom panel). Analysis of the averaged optical intensities of MMP‐9 and TGF‐β signals in the tested specimens identified significantly higher value in the groups of malignant tumour (Figure 1B, left). Further evaluations of the averaged optical intensities of MMP‐9 and TGF‐β signals according to the tumour malignancy illustrated higher values in the group of high malignancy than those in the groups of medium and low malignancy (Figure 1B, right). It implies again remarkable increases in expressions of MMP‐9 and TGF‐β in canine breast cancers, which coincidental well with the tumour malignancy.

**Table 2:** Immunohistochemical assays for matrix metalloproteinase (MMP)‐9 and transforming growth factor beta (TGF‐β) in 34 malignant breast cancers and 18 benign breast tumours

MMP‐9	Malignant			Benign		
	TGF‐β					
	Positive (%)	Negative (%)	Total (%)	Positive	Negative	Total (%)
Positive	24	5	29 (85.3)	1	1	2 (11.1)
Negative	3	2	5 (14.7)	3	13	16 (88.9)
Total	27 (79.4)	7 (20.6)	34 (100)	4 (22.2)	14 (77.8)	18 (100)

### 3.2 Transient expression of MMP‐9 in the breast cancer cell lines increases the cell abilities of colony formation and migration

To test the potential effect of MMP‐9 on the biological features of the cultured breast cancer cell lines, a eukaryotic expressing plasmid for the full‐length mouse MMP‐9 was constructed and transfected into cell lines 4T1, MDA‐MB‐231 and MCF7, respectively. Cells were harvested, and the expressions of exotic MMP‐9 were evaluated by Western blots. A roughly 100 kDa‐lager positive band was detected in the cells receiving pMMP‐9‐HA, but not in the cells receiving only the blank vector or mock control (Figure S1). No obvious difference in the expressing amount of exotic MMP‐9 was observed among the three tested cell lines. Besides, another specific band, migrating at the position of 78 kDa, was also detected in all cellular lysates regardless of receiving pMMP‐9‐HA or not, which represented endogenous MMP‐9 (Figure S1). It suggests the transient transfection of the constructed plasmid pMMP‐9‐HA into those three cell lines efficiently induces the expression of exotic MMP‐9.

**Figure 2:**
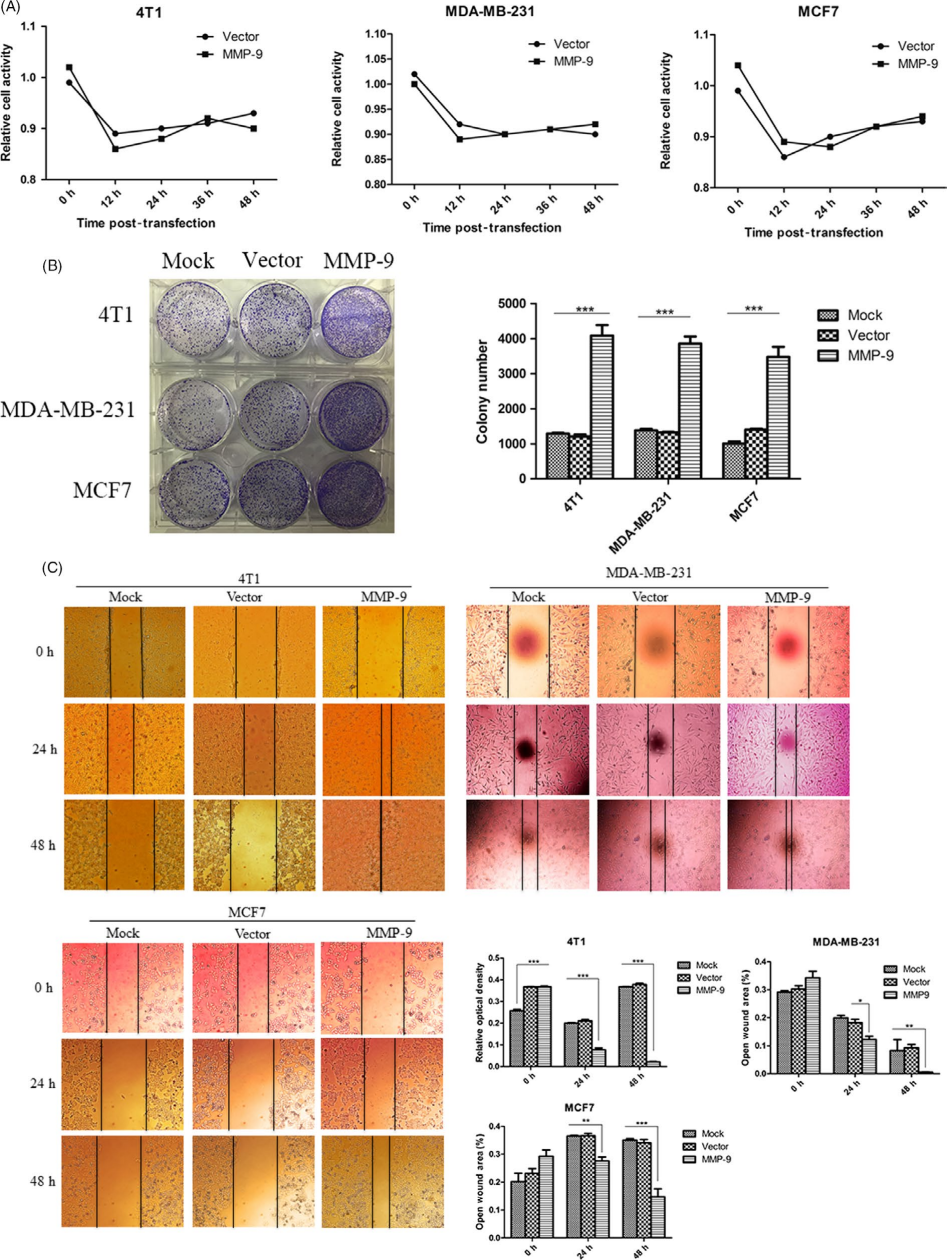
Evaluation of the malignancy of the cells after receiving pMMP‐9‐HA. A, Cell viability assays. Cells were assayed with commercial Cell Counting Kit‐8 kit at various time points after transfection. Each test was repeated for three times. Graphical data denote mean ± SD. B, Colony formation. The abilities of colony formation of the cells were evaluated 12, 24 and 48 h post‐transfection. C, Migration assay. The abilities of migration of the cells were tested 24 and 48 h post‐transfection. The black vertical lines in the images define the open wound areas. The average open wound areas of various preparations are indicated below. Each test was repeated for three times. Graphical data denote mean ± SD. Statistical analysis was performed using two‐way ANOVA. ***P < 0.001, **P < 0.01 and *P < 0.05 were considered significantly different

To see the possible influence of the transiently expressed MMP‐9 on cell biology, the viabilities of the different cell lines receiving plasmid pMMP‐9‐HA or blank vector were analysed by commercial Cell Counting Kit‐8 (CCK‐8) kit. As shown in Figure 2A, all three cell lines receiving plasmid pMMP‐9‐HA or blank vector illustrated similar dynamic curves of cell viability, that the viabilities dropped down markedly in the preparation of 12 hours post‐transfection representing the acute shock of the transfection reagent for the cells, and raised gradually but sustainably in the preparations of the subsequent checkpoints. It highlights that under our experimental condition and the time of observation, transient expression of exotic MMP‐9 does not affect cell viability.

To test the potential changes in some carcinoma‐associated characteristics of the cell lines expressing exotic MMP‐9, the cell abilities of colony formation were measured. Obviously, much more colonies were counted in the cell plates transiently transfected with plasmid pMMP‐9‐HA than those of mock control and vector control (Figure 2B), showing statistical difference. Wound assays also identified much narrower blank areas between two cell groups in the plates receiving plasmid pMMP‐9‐HA for 24 and 48 hours, indicating increased migrating abilities of the cells expressing exotic MMP‐9 (Figure 2C). Besides, no significant differences in colony formation and migrating ability were figured out among the preparations of those three tumour cell lines.

### 3.3 Overexpression of MMP‐9 promotes epithelial‐mesenchymal transition (EMT) of the cultured breast cancer cells

**Figure 3:**
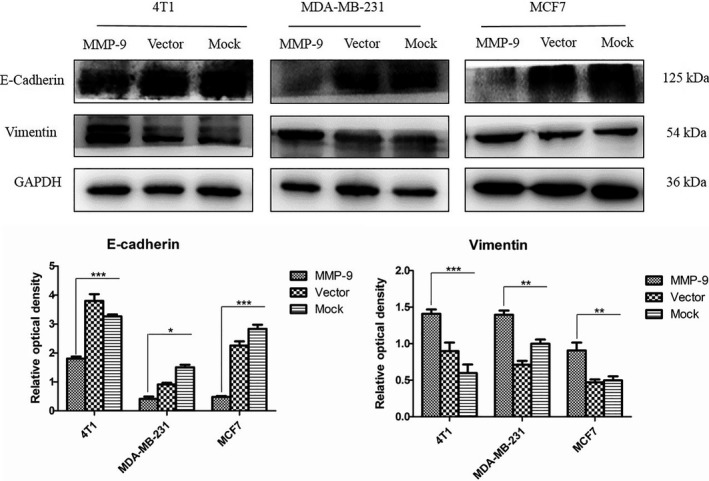
Analyses of the biomarkers for epithelial‐mesenchymal transition in the cells transfected with pMMP‐9‐HA. Western blots for E‐cadherin and vimentin. Cells were harvested 48 h post‐transfection. Quantitative assays of the relative grey values of E‐cadherin and vimentin after normalized with the data of GAPDH are shown. Each test was repeated for three times. Graphical data denote mean ± SD. Statistical analysis was performed using two‐way ANOVA. ***P < 0.001, **P < 0.01 and *P < 0.05 were considered significantly different

To address the possible alterations of the essential agents related to EMT in the cells after overexpression of MMP‐9, the levels of two proteins, E‐cadherin and vimentin, in three cell lines were tested. Western blots identified that compared with the observations in the mock and vector controls, the signals of E‐cadherin in the cells transiently expressing exotic MMP‐9 were weaker, while that of vimentin became stronger (Figure 3). Quantitative assays of the average grey values after normalized with the individual data of GAPDH proposed statistical differences in all tested cell lines. It seems that overexpressing MMP‐9 in the cultured breast cells may efficiently induce EMT.

### 3.4 Overexpression of MMP‐9 induces the release of the activated form of TGF‐β in the cultured breast cancer cells

**Figure 4:**
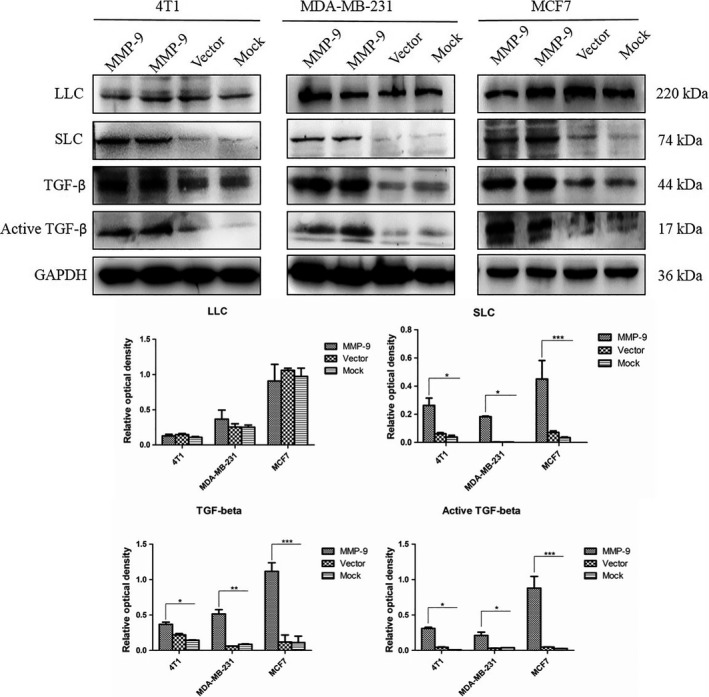
Alterations of the cellular transforming growth factor beta (TGF‐β) and its isoform (complexes) in the cells transfected with pMMP‐9‐HA. Western blots for TGF‐β. Cells were harvested 48 h post‐transfection. Quantitative assays of the relative grey values of various TGF‐β isoforms after normalized with the data of GAPDH are shown. Each test was repeated for three times. Graphical data denote mean ± SD. Statistical analysis was performed using two‐way ANOVA. ***P < 0.001, **P < 0.01 and *P < 0.05 were considered significantly different

Transforming growth factor beta homodimer interacts with other cellular proteins and formed small latent complex (SLC) and large latent complex (LLC), which were in the inactive state.^23^ MMP‐9 and MMP‐2 are known to cleave latent TGF‐β. To see whether overexpression of MMP‐9 in the breast cancer cell lines could release TGF‐β from the LLC from the matrix, various cellular lysates were subjected to Western blots. As illustrated in Figure 4, all tested breast cancer cells maintained baseline expressions of TGF‐β (about 44 kDa) and other isoforms, which were unchanged when transfected with blank vector. The signals of the cellular TGF‐β, as well as SLC (about 78 kDa) and activated form of TGF‐β (about 17 kDa), became much stronger in the cells transiently expressing, whereas that of LLC (about 220 kDa) maintained almost unchanged. Quantitative assay verified significantly higher grey values of TGF‐β, SLC and activated TGF‐β in the cells overexpressing MMP‐9. Those results indicate that overexpression of MMP‐9 in breast cancer cells promotes the proteolysis process of TGF‐β precursor.

### 3.5 Overexpression of MMP‐9 induces upregulation for the expression and phosphorylation of SMAD proteins

**Figure 5:**
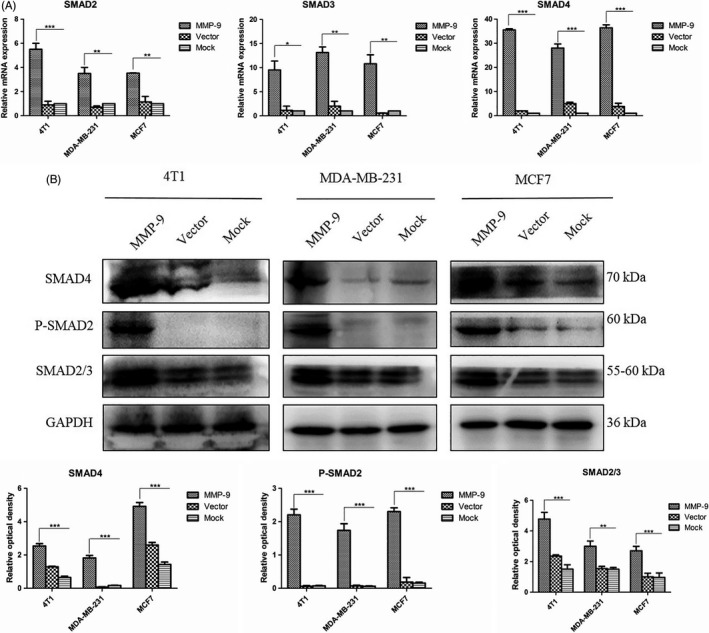
Analyses of the changes in cellular SMADs in the cells transfected with pMMP‐9‐HA. Cells were harvested 48 h post‐transfection. A, qRT‐PCR assays. The total RNA was prepared, and the transcriptional levels of various *SMAD* genes were evaluated with the individual qRT‐PCRs. *Y*‐axis represents the values of 2^−ΔΔCT^. Each test was repeated for three times. Graphical data denote mean ± SD. B, Western blots. The cellular levels of various SMADs were determined with the individual Western blots. Quantitative assays of the relative grey value of each SMAD after normalized with the data of the individual GAPDH are shown. Each test was repeated for three times. Graphical data denote mean ± SD. Statistical analysis was performed using two‐way ANOVA. ***P < 0.001, **P < 0.01 and *P < 0.05 were considered significantly different

To test the possible influence on the expression and phosphorylation of SMAD proteins, three breast cancer cell lines were transiently transfected with plasmid pMMP‐9‐HA for 48 hours. The harvested cells were subjected to total RNA extraction and cellular lysate preparation. qRT‐PCR assays for the cellular genes *SMAD2, SMAD3 and SMAD4* showed significantly increased transcriptions in the cells overexpressing MMP‐9, varying from 3.5‐ to 5.5‐fold increase in *SMAD2*, 9.5‐ to 13‐fold increase in *SMAD3* and 27‐ to 35‐fold increase in *SMAD4* (Figure 5A). Western blots revealed much stronger bands of SMAD2/3 and SMAD4 in all three cell lines receiving plasmid pMMP‐9‐HA, showing significantly statistical differences compared with those of mock and vector control (Figure 5B). Moreover, the levels of the phosphorylated form of SMAD2 (p‐SMAD2) were evaluated by Western blots. Compared with weak signals in mock and vector control cells, the p‐SMAD2 signals in all tested cells overexpressing MMP‐9 were much stronger, showing significantly increased in the quantitative assays of the average grey values (Figure 5B). These data indicate that overexpression of MMP‐9 in the cultured breast cancer cells not only upregulates remarkably the expressions of the cellular SMAD2, SMAD3 and SMAD4, but also enhances the phosphorylation for SMAD2.

### 3.6 Inhibition of TGF‐β or MMP‐9 activity abolishes the enhancing effect of overexpression of MMP‐9 on SMAD signal pathway in the cultured breast cancer cells

**Figure 6:**
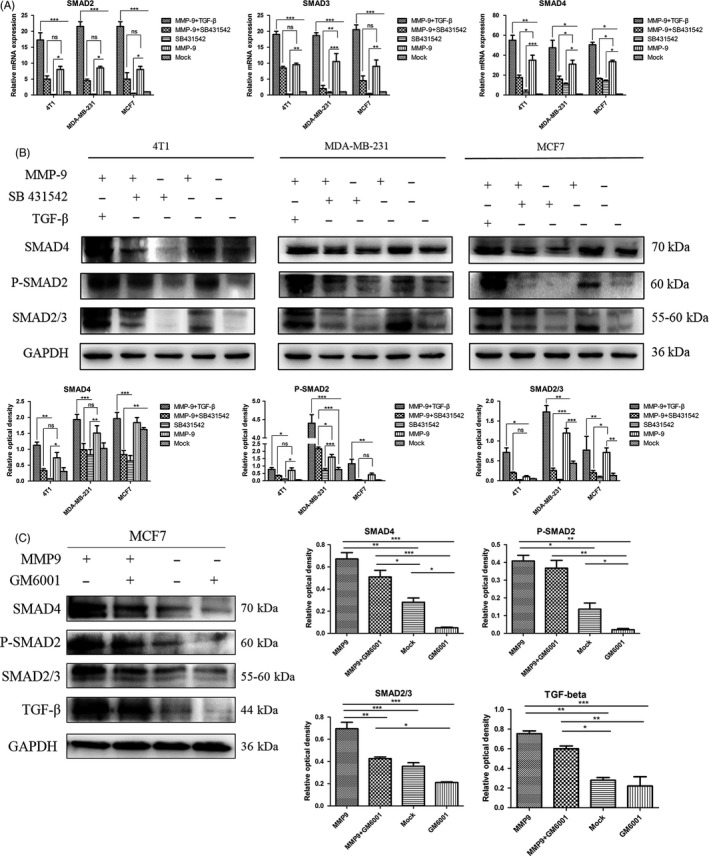
Influences of transforming growth factor beta (TGF‐β) inhibitor and matrix metalloproteinase (MMP)‐9 inhibitor on the cellular levels of various SMADs in the cells transfected with pMMP‐9‐HA. A, B, Assays of the influence of TGF‐β inhibitor. Cells were harvested 48 h post‐transfection. The recombinant TGF‐β and/or the inhibitor SB 431542 were added to the cell cultures 12 h after transfection. A, qRT‐PCR assays. *Y*‐axis represents the values of 2^−ΔΔCT^. Each test was repeated for three times. Graphical data denote mean ± SD. B, Western blots. The cellular levels of various SMADs were determined with the individual Western blots. Quantitative assays of the relative grey value of each SMAD after normalized with the data of the individual GAPDH are shown. Each test was repeated for three times. Graphical data denote mean ± SD. C, Assay of the influence of MMP‐9 inhibitor. The pMMP‐9‐HA and/or MMP inhibitor GM6001 were added to MCF7 cells and harvested 48 h post‐transfection. The cellular levels of various SMADs and TGF‐β were determined with the individual Western blots. Quantitative assays of the relative grey value of each SMAD and TGF‐β after normalized with the data of the individual GAPDH are shown right. Statistical analysis was performed using two‐way ANOVA. ***P < 0.001, **P < 0.01 and *P < 0.05 were considered significantly different

SB431542 is a selective inhibitor of the TGF‐β1 receptor ALK5, whose molecule weight is 384.39.^24^ To address the association of overexpressing MMP‐9 with the activation of TGF‐β/SMAD signal pathway in various breast cancer cells, SB 431542 (10 μmol/L) was added into the cultured cells, alone or together with MMP‐9 or MMP‐9/TGF‐β. qRT‐PCR assays (Figure 6A) found that the transcriptions of genes of *SMAD2, SMAD3 and SMAD4* in the cells treated with SB431542 alone were similar as that of mock control, even slightly lower. Transfection of plasmid pMMP‐9‐HA together with addition of TGF‐β (50 pmol/L) into the cells induced highest level of specific mRNA transcriptions. Compared with the data of transfection of plasmid pMMP‐9‐HA showing increased levels of transcriptions of those three *SMAD* genes, the transcriptional levels of the tested three *SMAD* genes in the cells receiving pMMP‐9‐HA and SB431542 were relatively lower. SMAD‐specific Western blots of the cellular lysates revealed the similar profiles (Figure 6B). The cells overexpressing MMP‐9 and exposed to TGF‐β simultaneously contained remarkably strong SMAD2/3, SMAD4 and p‐SMAD2. Most of the preparations treated with pMMP‐9‐HA and SB431542 showed the relatively lower levels of the tested SMAD proteins than those of the cells receiving pMMP‐9‐HA alone. Similarly, the levels of SMAD proteins in the cells treated with SB431542 alone were low, which were comparable with that of the mock or even lower in some reactions. It highlights that the enhancing effect of overexpression of MMP‐9 on SMAD signal pathway depends on, at least partially, the activation of TGF‐β.

To get the direct evidence of inhibition of MMP‐9 activity on the expressions of SMADs, GM6001, an inhibitor for MMPs, was added into the cell line MCF7. Western blot showed that in the presence of GM6001, the cellular levels of TGF‐β and SMADs (SMAD4, SMAD2/3 and p‐SMAD2) were clearly lower, regardless of receiving plasmid pMMP‐9‐HA (Figure 6C, the left two lanes) or not (the right two lanes). It indicates that activations of TGF‐β and SMAD signal pathway in breast cancer cell line can be blocked by inhibition of MMP‐9 activity.

### 3.7 Inhibition of TGF‐β activity in breast cancer cells blocks the MMP‐9‐induced enhancement on invasion

**Figure 7:**
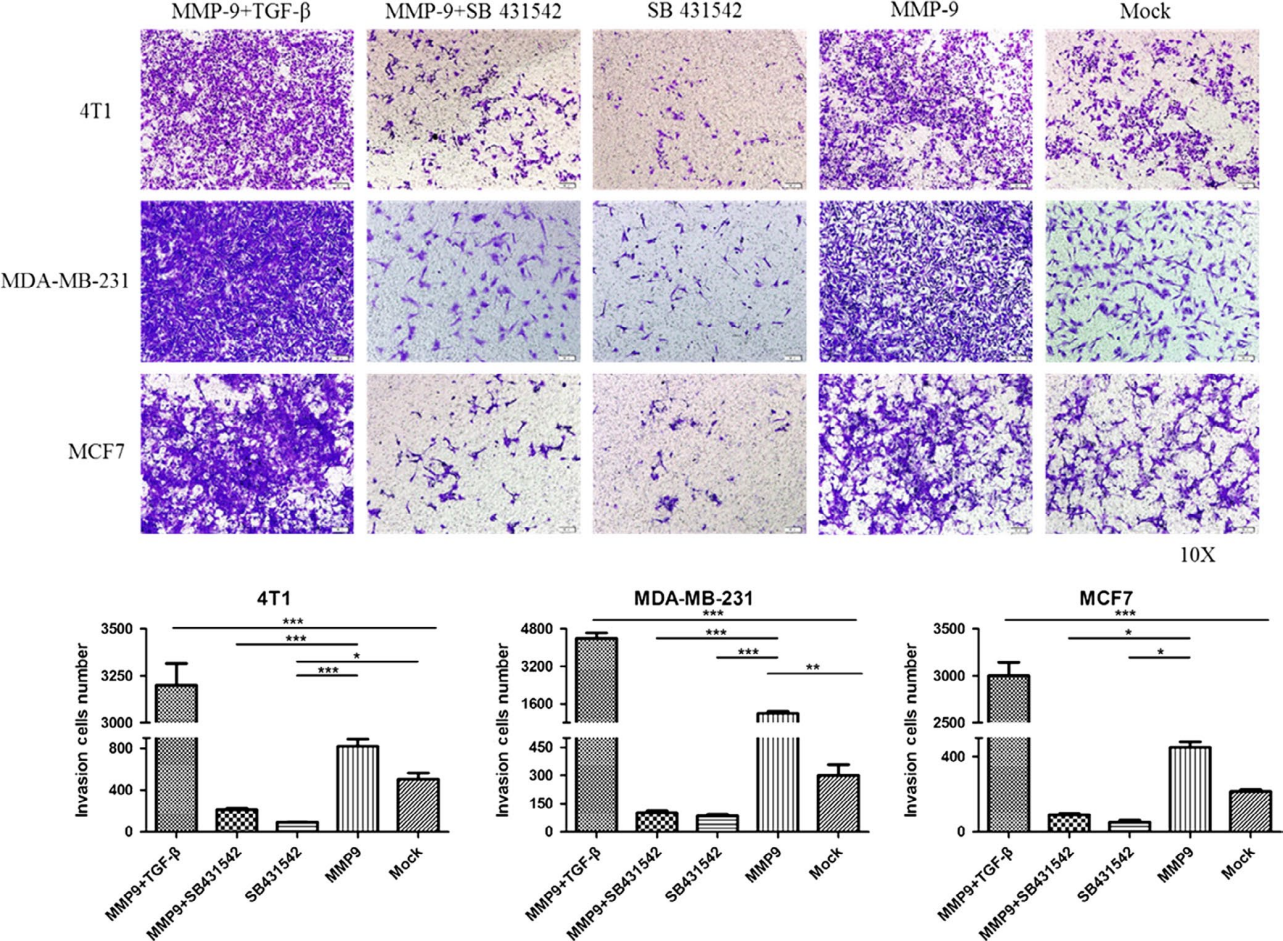
Influences of the transforming growth factor beta (TGF‐β) inhibitor on the invasion abilities of the cells transfected with pMMP‐9‐HA. Representative images of cells in the lower side of the filter. Cells were exposed to recombinant TGF‐β and/or the inhibitor SB 431542 12 h post‐transfection. The migration of cells to the lower side of the filter was determined by crystal violet staining. Each testing group contained at least three independent wells. Statistical analysis was performed using one‐way ANOVA. ***P < 0.001, **P < 0.01 and *P < 0.05 were considered significantly different

To test the influence of overexpressing MMP‐9 in breast cancer cells on their invasion abilities and figure out the relevant molecular mechanism, assays of transwell filters were conducted. Compared with the observations in the mock controls, more numbers of cells were seen in the preparations transfected with pMMP‐9‐HA, indicating that overexpression of MMP‐9 increases the invasion abilities of various breast cancer cells (Figure 7). Transfection with pMMP‐9‐HA together with exposure to TGF‐β induced even more numbers of cells migrating to the lower side of the filter (Figure 7). In contrary, the numbers of the cells migrating to the lower side of the filter were obviously less in the preparations receiving pMMP‐9‐HA but exposing to SB431542, which looked similar as the mock (Figure 7). It suggests the enhanced invasion ability of the breast cancer cells by overexpressing MMP‐9 relies largely on the activation of TGF‐β/SMAD signal pathway.

## 4 DISCUSSION

In this study, we have confirmed again markedly increased expressions of MMP‐9 and TGF‐β in the malignant tissues of 34 canine breast carcinomas. The expressing levels of MMP‐9 and TGF‐β in the IHC assays, displayed as the distribution and intensity of the positive signals, seem not to be related with the onset ages and tumour sizes of the diseased dogs, but show close association with the tumour malignancy. In line with our observation, a study of human breast cancers has also proposed no correlations of abnormal MMP‐9 and MMP‐2 with the age of the patient and tumour size, but significant correlations with the lymphatic metastasis and clinical stage.^25^ All enrolled canine breast cancers in this study are infiltrative ones. Higher levels of MMP‐9 and MMP‐2 have been repeatedly described in the malignant tissues of the patients with infiltrative breast cancer and lymph node metastasis.^11^, ^26^, ^27^, ^28^

Our data here illustrate that transient overexpression of MMP‐9 in the breast cancer cell lines strongly enhances the cellular malignant characteristics in vitro, such as the cell colony formation, migration and EMT. Along with these changes, an alteration of the cellular TGF‐β profiles in the cells overexpressing MMP‐9 is noticed. It is known that TGF‐β is synthesized as precursor molecules containing a propeptide region in addition to the TGF‐β homodimer. TGF‐β homodimer interacts with a latency‐associated peptide (LAP) and forms SLC. SLC remains in the cell until it binds with another protein called latent TGF‐β‐binding protein (LTBP) and forms a large complex LLC that gets secreted to the extracellular matrix (ECM).^23^, ^29^ Our data here have revealed that not only the activated form of TGF‐β but also cellular TGF‐β and SLC increase in the cells expressing MMP‐9. It indicates that overexpression of MMP‐9 in the transfected cells may not only promote the proteolysis of TGF‐β, but also influence the expression of TGF‐β. The observations of stronger TGF‐β deposits in the MMP‐9‐positive areas in the malignant tissues also propose the probable correlation between those two elements during carcinogenesis. We also find that the levels of LLC maintain almost unchanged, even slightly declined, after challenge of MMP‐9. As LLC is usually secreted into ECM, the lysates for LLC are carefully prepared without PBS washing in order to avoid the potential loss of LLC during the harvesting process. Those diversity profiles of TGF‐β complexes in the breast cancer cells during overexpression of MMP‐9 deserve further study.

The activated TGF‐β binds to TGF‐β type II receptor (TGF‐βRII) and further phosphorylates TGF‐β type I receptor (TGF‐βRI). The binding of TGF‐βRII and TGF‐βRI promotes the downstream signalling by phosphorylating cytoplasm mediators, SMAD2 and SMAD3, which complex with SMAD4 and translocate into the nucleus and regulate the transcriptions of target genes.^30^, ^31^ Our data here have proposed the evidences that MMP‐9 in the breast cell lines regulates the phosphorylation of SMAD2/3 and increases the cellular levels of SMADs. Blocking the binding of TGF‐β to its receptor by special inhibitor attenuates MMP‐9‐induced phosphorylation of SMAD2/3 and the increases in SMADs in the breast cancer cells. It depicts a clear reactive chain that MMP‐9 in cancer cell lines sufficiently activates TGF‐β/SMAD signalling. The changes in cellular SMADs induced by overexpression of MMP‐9 are mediated via TGF‐β.

The functions of the TGF‐β/SMAD signalling pathway in cancer seem to be paradoxical. Such phenomenon has been described for long time.^32^ The suppressive role of this signalling in cancers reflects in inhibition on tumour formation and inducement of growth arrest and apoptosis.^33^, ^34^ On the other hand, TGF‐β/SMAD signalling shows the activity to promote tumour progression and metastasis, by inducing angiogenesis, inflammation and EMT.^35^, ^36^ Yoshida and the colleagues 31 have concluded, based on the reviews of the publications, that TGF‐β acts as a growth suppressor in normal epithelial cells after transient Ras activation. During the transition from human benign tumours to carcinomas in situ, tumours with Ras‐activating mutations, TGF‐β gradually loses growth inhibitory effects. The inhibitor for TGF‐β1 receptor in this study sufficiently reverses the TGF‐β‐ and/or MMP‐9 induced the increased invasive abilities of the breast cell lines supplies further evidences supporting that TGF‐β/SMAD signalling increases malignancy of the cultured breast cancer cells. One may assume that the activated TGF‐β/SMAD signalling induced by overexpression of MMP‐9 may also promote the progression and probably metastasis of canine breast cancers.

We have also noticed that MMP‐9 overexpression in breast cancer cell lines increases the transcriptions of SMADs. The exact mechanism remains unknown. MMP‐9 and its family members seem not to function directly as transcriptional activator or regulator. However, TGF‐β, after activation, regulates the expressions of a number of target genes via phosphorylation of SMAD2/3.^37^ During this pathway, Krüppel‐like factor 10 (KLF10) plays an essential role. KLF10, originally named TGF‐β‐inducible early gene 1, is a DNA‐binding transcriptional regulator containing a triple C2H2 zinc finger domain.^38^ Through binding to specificity protein 1 sites on the DNA and interacting with other transcriptional factors, KLF10 enhances or suppresses the expressions of numerous genes in many different types of cells. The expression of KLP10 is induced by phosphorylated SMAD2/3 complexed with SMAD4 after translocating to nucleus. KLF10 binds to the promoters of SMAD2 and TGF‐β1, and more importantly, it serves as a positive feedback loop for regulating TGF‐β signalling by inducing the expression of SMAD2.^39^ Based on this case, we assume that the MMP‐9 induced the transcriptional increases in SMADs in breast cancer cell lines may link with activation of TGF‐β/SMAD signalling and KLF10. Nevertheless, the exact molecular mechanism deserves further study.

**Figure 8:**
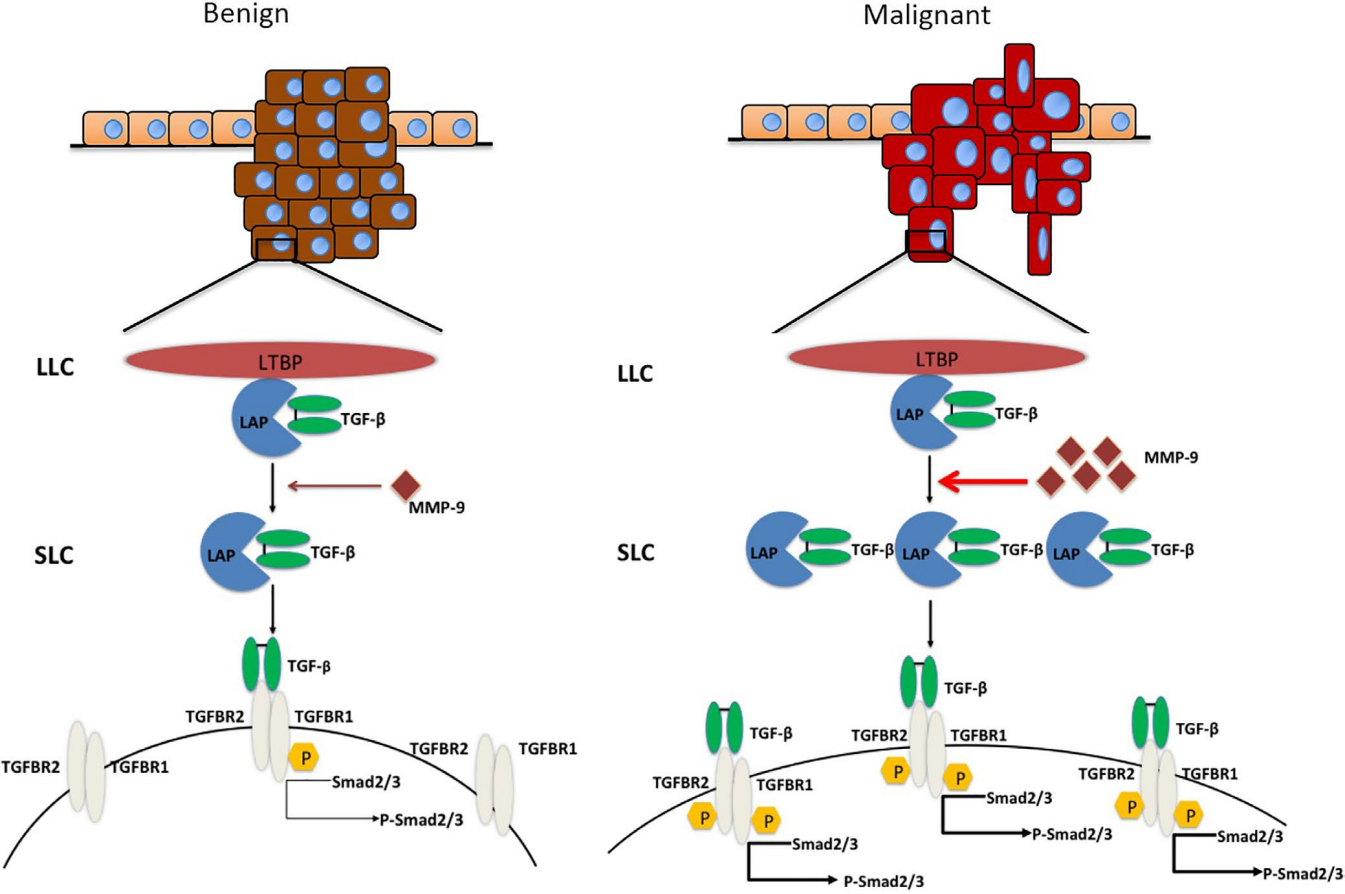
A hypothetical processing schema of the matrix metalloproteinase (MMP)‐9‐mediated activation of transforming growth factor beta (TGF‐β)/SMAD pathway in malignant and benign tumours. In malignant tumour cells, more MMP‐9 molecules are expressed, which induce the more active releases of SLC and TGF‐β from the latent form and phosphorylation of TGF‐β receptors and SMAD2/3

In summary, more aggressive expressions of MMP‐9 and TGF‐β were detected in the malignant canine breast cancers. Overexpression of MMP‐9 in the breast cancer cell lines increased the malignancy in vitro, which is likely associated with the activation of TGF‐β/SMAD pathway. As illustrated in Figure 8, we hypothesize that in malignant breast cancers, more MMP‐9 are expressed, which leads to more releases of TGF‐β from its latent form. More activated TGF‐β further induces phosphorylation of TGF‐β receptor, activates the TGF‐β/SMAD signalling and eventually increases the malignancy of cancer.

## CONFLICTS OF INTERESTS

The authors declare no competing financial interests.
