# Supplementary figures and images for: Overexpression of matrix metalloproteinase‐9 in breast cancer cell lines remarkably increases the cell malignancy largely via activation of transforming growth factor beta/SMAD signalling
Source: Cell Prolif. 2019 Jul 2;52(5):e12633. doi: 10.1111/cpr.12633 (PMC6797518; doi:10.1111/cpr.12633)

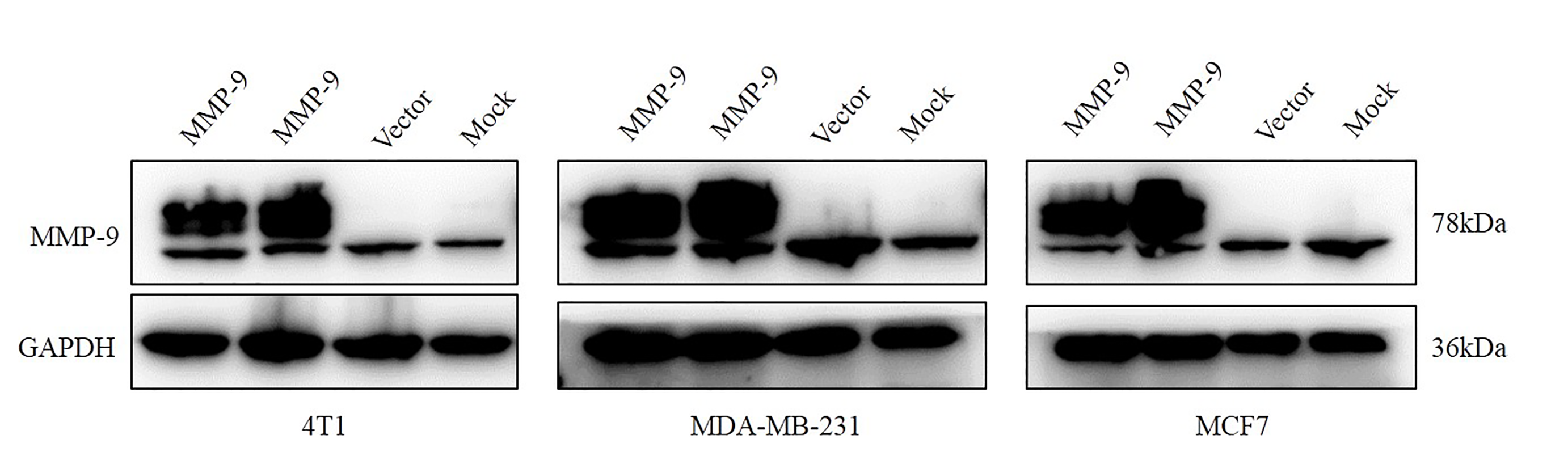

Supplement: Supplementary file 1 [file CPR-52-e12633-s001.tif]
